# History of pancreatic surgery in Japan: Respect to the Japanese pioneers of pancreatic surgery

**DOI:** 10.1002/ags3.12320

**Published:** 2020-03-04

**Authors:** Hiroki Yamaue

**Affiliations:** ^1^ Second Department of Surgery Wakayama Medical University Wakayama Japan

**Keywords:** mesenteric approach, pancreatic surgery, pancreatoduodenectomy, pioneer, portal vein resection

## Abstract

The first report of pancreatoduodenectomy was the abstract of Japan Surgical Society in 1946 by Kuru, followed by a publication by Yoshioka (Geka, 1950). The first report of total pancreatectomy was done by Honjo in 1950 (Shujutsu). Thus, the history of pancreatic surgery in Japan dawned in the 1950s. From 1970 to 1980, the American surgeon Fortner had reported the drastic concept of regional pancreatectomy with extensive dissection of vessels and connective tissues around the pancreas. A lot of Japanese surgeons were influenced by this concept and attempted to perform the extensive surgery of pancreatic cancer, especially the Japanese pioneers who had investigated the clinical benefits of extensive surgery with dissection of nerve plexus and lymph nodes around the superior mesenteric artery. Then, Japanese surgeons had a great attention for limited resection of the pancreas for borderline malignancies, and Japan was the number one country for pancreatic surgery for all pancreatic diseases, from advanced pancreatic cancer to borderline malignancies. The next step for these pioneers was how to reduce morbidities after pancreatic surgery, especially pancreatoduodenectomy. Due to the effects of technical development, drain management, and nutritional consideration, the incidences of pancreatic fistula and delayed gastric emptying decreased dramatically in the past 10 years. Moreover, the development of chemotherapeutic drugs has provided a new era of conversion surgery, similar to esophageal surgery, and one should pay great attention to more aggressive surgery, including distal pancreatectomy with en bloc celiac axis resection (DP‐CAR). Thus, we have to inherit the passion and mentality of the Japanese pioneers of pancreatic surgery and develop safer and more secure surgical techniques to reduce the morbidities and elongate the survival of pancreatic cancer patients.

## 1 INTRODUCTION

Pancreatic cancer is definitely a most dismal malignant disease and the survival rate has been still poor, despite the extensive efforts of pancreatic surgeons and other medical staff. In terms of surgical treatment of patients with pancreatic cancer, one should be aware of the high morbidity and mortality rates compared to other surgical procedures, especially high incidence of morbidities including pancreatic fistula, intraabdominal abscess, and delayed gastric empty. In this review article, the history of pancreatic surgery in Japan is examined in a retrospective review in order to understand the advances of the Japanese pioneers of pancreatic surgery.

Moreover, it is important that we inherit the passion and mentality of these Japanese pioneers of pancreatic surgery and develop safer surgical techniques to reduce the morbidities and elongate the survival of pancreatic cancer patients with the combination of other treatment modalities.

## 2 GLORIOUS PIONEERS OF PANCREATIC SURGERY IN WESTERN COUNTRIES

Great attempts for radical resection of pancreatic cancer by pancreatic surgery pioneers in Western countries have compromised the development of safer pancreatic surgery. Schnelldorfer et al have reported the history of pancreatic surgery in Western countries in their 2008 report titled " Forgotten pioneers of pancreatic surgery: Beyond the favorite few." Here they clearly show the great history of pancreatic surgery, shining light on modern literatures and also on a piece of lost history in pancreatic surgery.^1^

**Figure 1:**
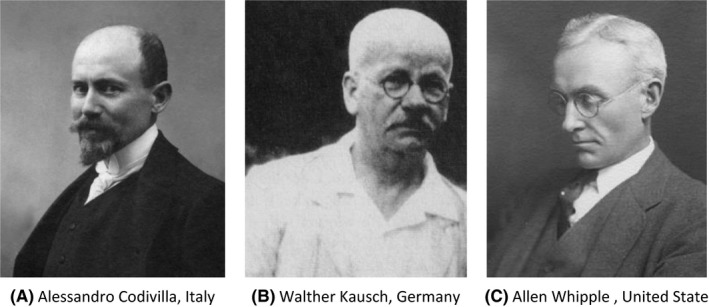
Pioneers of pancreatic surgery in western countries. First pancreatoduodenectomy was performed by Italian surgeon, Codivilla (A). First modern surgery was done by Kausch (B) in Germany and also by Whipple in United State (C), and referred from reference #1. Pancreatoduodenectomy has been called Kausch's operation or Whipple's operation nowadays

One should recognize as a landmark date in the history of pancreatic resection as 9 February 1898 when Alessandro Codivilla (Figure 1A) of Imola, Italy, performed the first pancreatoduodenectomy. In 1908, Sauve reported the first operation by Codivilla, referring to the statistical report of the hospital of Imola.^2^ Many publications have cited this reference as the first pancreatoduodenectomy; however, the original report was lost and unavailable despite great interest in the history of pancreatic surgery.^1^

The evolution toward modern pancreatic surgery was due to Walther Kausch (Figure 1B). Kausch performed the first modern pancreatoduodenectomy in 1909.^3^ The patient was a 49‐year‐old male with weight loss and jaundice as chief complaints, and the patient received a two‐stage operation, with anastomosis to reduce the jaundice as the first operation, and pancreatoduodenectomy as a second operation. The patient had severe bile leakage and pancreatic fistula and died 9 months after surgery due to septic shock caused by cholangitis.

In the United States, Whipple (Figure 1C) reported the surgical technique of pancreatoduodenectomy as a modern surgery in a report titled “Treatment of carcinoma of the ampulla of vater” in 1935.^4^

**Figure 2:**
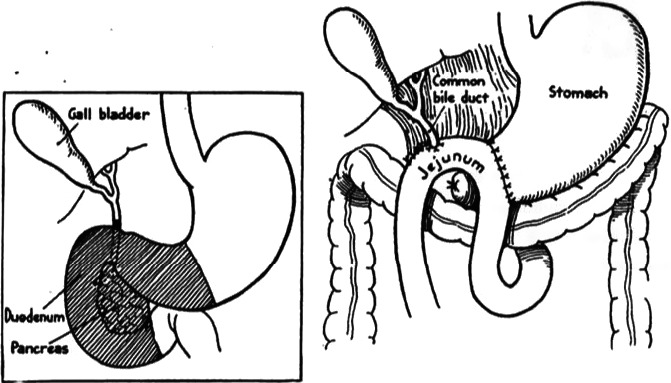
Whipple's one stage radical pancreatoduodenectomy with antecolic gastrojejunostomy and implantation of the common duct into the jejunum. The patient had a dilated pancreatic duct which was ligated and the pancreas was not anastomosed with jejunum or stomach. This figure was referred from reference #5

Whipple had performed a two‐stage operation composed of a first‐stage operation of gastroenterostomy and cholecystogastrostomy, and a second‐stage operation of resection of pancreatic head followed by reconstruction at 3 to 4 weeks after the first‐stage operation.^4^ Then, Whipple had developed a one‐stage radical operation for a 53‐year‐old female patient. At that time, Whipple ligated the dilatated pancreatic duct and had never performed the anastomosis of pancreatic remnant with jejunum or stomach (Figure 2). Moreover, Whipple and his colleagues had developed a superior operation with anastomosis of the remnant pancreas including pancreatojejunostomy, reaching the completion of, what is now known as, the standard operation of a pancreaticoduodenectomy.^5^

## 3 PIONEERS OF PANCREATIC SURGERY IN JAPAN

In Japan, Kuru was the first surgeon who performed pancreaticoduodenectomy in 1943 and presented about this operation in 1946 at the annual meeting of the Japan Surgical Society.^6^ Then, Yoshioka published the first article on pancreaticoduodenectomy in 1947, written in Japanese.^7^ The first English article of pancreaticoduodenectomy in Japan was reported by Imanaga who successfully developed a physiological Billroth I anastomosis of the gastric remnant with jejunum.^8^ Thus, Japan has a history of pancreatic surgery from 1940s, and established the reconstruction methods during 1950s.

The first annual meeting of the Japanese Society of Gastroenterological Surgery (JSGS) was held by Yamagishi in 1968 in Yokohama, and the main topics were gastric surgery including surgery for gastric ulcer, especially selective vagotomy for gastric ulcer. It was the 3rd annual meeting of the JSGS in Okayama in 1971 when Tanaka presented on pancreatic surgery. The title of the symposium was “Surgical treatment of malignant tumor in pancreas and bile duct,” and Japanese pioneers of pancreatic surgery presented their data, including diagnosis, bypass operation, and radical operation of periampullary tumors.

The 13th annual meeting of JSGS was held by Yokoyama in Kumamoto in 1979, and the main cine‐symposium was how to dissect the connective tissues around superior mesenteric artery (SMA). Considering the development and innovation of pancreatic surgery in Japan, one should divide the history into four categories: the development of surgical procedures; how to maintain quality of life after pancreatic surgery; multidisciplinary treatment; and the future perspective of this surgery.

### 3.1 Extended pancreatic surgery in Japan

First of all, many Japanese pancreatic surgeons had a great influence, such as Joseph G. Fortner at Memorial Sloan‐Kettering Cancer Center. Fortner proposed a very unique, extended technique named “regional pancreatectomy” as a radical surgery for pancreatic cancer. Regional pancreatectomy was one of the most extended pancreatic surgeries, with resection of portal vein, common hepatic artery, and SMA to decrease the incidence of remnant tumor cells in surgical fields.^9^

In Japan, Ishikawa is a great pioneer of extended pancreatic surgery and he has performed complete dissection of lymphatic and connective tissues around the common hepatic artery and SMA; the benefit of this extensive surgery is clearly indicated by the better survival of patients with tumor size less than 4 cm compared to conventional and standard dissection.^10^ However, clinical significance of extended pancreatic surgery has not been proved by five randomized controlled trials (RCT), including one study from Italy, two from the USA, one from Japan, and one from Korea. Nimura et al had performed multicenter RCT that compared standard vs extended lymph node dissection for patients with pancreatic cancer, but unfortunately extended lymphadenectomy did not benefit long‐term survival with high morbidity and poor quality of life outcomes.^11^ What are the clinical benefit of dissection of connective tissues around SMA? The survival rate of patients with perineural invasion was low compared to patients without invasion, thus Nagakawa argued for the total clearance of connective tissues around SMA.^12^

### 3.2 Mesenteric approach as an innovative surgical procedure

**Figure 3:**
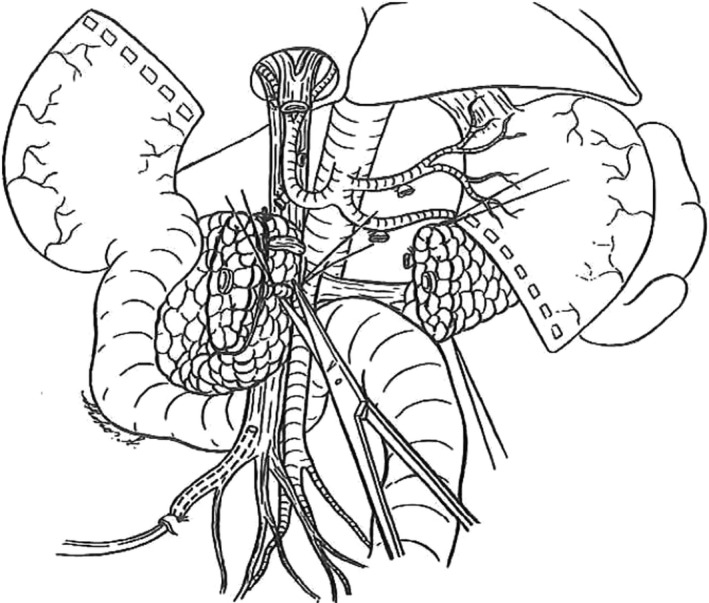
Mesenteric approach for pancreatic head cancer The feeding artery including inferior pancreatoduodenal artery was ligated at the early phase of the operation to reduce the blood loss, and complete dissection of lymph nodes and connective tissues around SMA was possible by this technique. This figure was referred from reference #15 by Nakao et al

**Figure 4:**
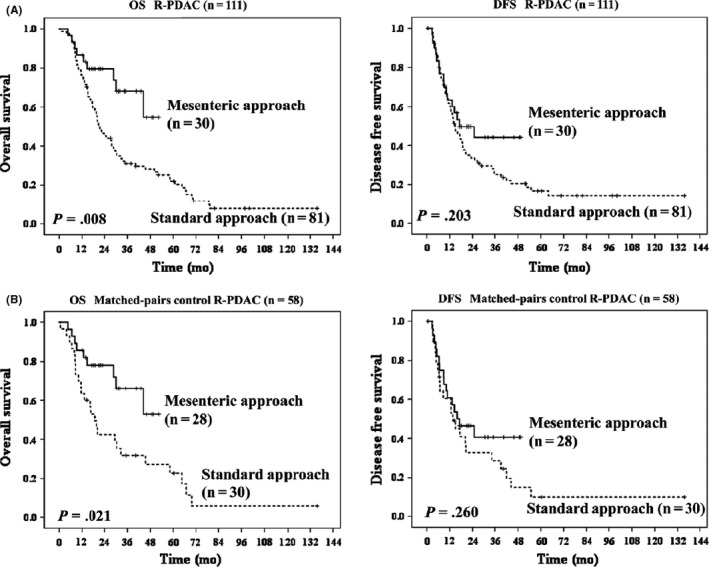
Clinical benefit of mesenteric approach in patients with resectable pancreatic cancer. (A) Overall survival in the resectable pancreatic cancer patients (R‐PDAC) was longer in mesenteric approach, and (B) by matched‐pairs comparative analysis of survival outcomes between the mesenteric group and the conventional group, overall survival in R‐PDAC was longer in the mesenteric approach. This figure was referred from reference #16 by Hirono et al

The most important issue for pancreatic surgery is to achieve remnant cancer‐cell‐free status after surgery. To accomplish this situation, a special approach has been proposed from Heidelberg named “The artery first approach for resection of pancreatic head cancer,” and this term has spread worldwide.^13^ This technique provides the following merits: judgement of resectability at early phase of the operation; reduction of blood loss during operation by early ligation of feeding arteries including inferior pancreatoduodenal artery; complete dissection of lymph nodes and connective tissues around SMA; and allowance of isolated pancreatectomy with non‐touch isolation technique.^14^ However, when we look at the history of the literature, the first surgeon to perform the artery‐first approach was Nakao at Nagoya in 1983.^15^ Since Nakao proposed the concept of the mesenteric approach (Figure 3), many surgeons have tried to follow this technique; however, there have been only small studies that have only reported on the feasibility of the mesenteric approach, and evidence for survival benefit has not been reported so far. Wakayama Medical University first reported the oncological benefits of mesenteric approach in terms of long‐term survival using matched‐pair comparative analysis of our case series.^16^ As shown in Figure 4, the overall survival was much longer in the mesenteric group compared to the conventional group. However, this study has several limitations, especially the fact that it includes a retrospective cohort at a single institute. Therefore, we have decided to perform the multicenter RCT "MAPLE‐PD trial" in Japan to compare the mesenteric and conventional approach in terms of long‐term survival.^17^

### 3.3 Portal vein resection during pancreaticoduodenectomy

One of the most important progressions in pancreatic surgery should be the advanced technique of portal vein resection. Pancreatic cancer tends to invade portal vein and superior mesenteric vein (SMV) and the surgeons are sometimes required to resect the portal vein and SMV. Moreover, we have to state that staging of the tumor increases if the tumor invades the portal vein and SMV (borderline resectable pancreatic cancer with invasion to portal vein; BR‐PV). In Japan, we have two representative articles from Osaka and Nagoya. First, Ishikawa clearly divided the patients into four categories depending on the degree of portal vein invasion; this is known as Ishikawa's criteria.^18^ Second, Nakao also classified four categories of portal vein invasion according to the computed tomography (CT) imaging with correlation to pathological grade of portal vein wall invasion.^19^ Both studies had similar results with poor prognosis for severe invasion to the portal vein.

Regarding the reconstruction of portal vein, end‐to‐end anastomosis is a usual technique; however, autologous venous graft should be used if the length of the portal vein is too long to reconstruct with the end‐to‐end fashion. In this situation, Miyazaki from Chiba demonstrated a unique surgical technique, that is, portal vein reconstruction at the hepatic hilus using a left renal vein graft.^20^ Moreover, Miyazaki's group have demonstrated that renal function is well maintained even after use of left renal vein graft for vascular reconstruction.^21^ What kind of autologous vein should be used for graft of portal vein reconstruction? We have reported that various kinds of autologous vein can be used including left renal vein, external iliac vein, and internal jugular vein.^22^ The indication of vein graft is considered regarding long‐term patency, and we proposed using the vein graft if the length of portal vein resection is over 5 cm in pancreas‐head resection and over 3 cm in pancreas‐body resection. Vein graft should be used not only for short‐term complications, but also for long‐term patency of portal vein and SMV.^22^

### 3.4 Distal pancreatectomy with en bloc celiac axis resection (DP‐CAR)

It was first reported by Nimura that a patient with locally advanced pancreas body cancer was operated with resection of celiac axis, that is, an application of Appleby's operation in gastric cancer surgery.^23^ In this operation, total gastrectomy was performed due to the resection of celiac axis. The first article where the stomach could be preserved was published from Tochigi by Hishinuma and Ogata in 1991,^24^ and they experienced two cases with distal pancreatectomy with celiac axis resection (DP‐CAR) and preserved the whole stomach for better quality of life after surgery. Indeed, Ogata et al never used the medical terminology "Appleby's operation" in this special operation with preservation of the stomach, because Appleby's operation was developed in gastric cancer surgery and should perform total gastrectomy.

**Figure 5:**
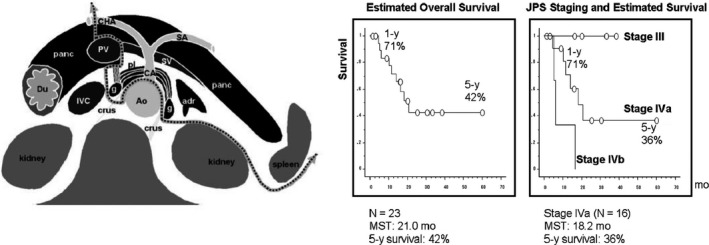
Distal pancreatectomy with en‐bloc celiac axis resection (DP‐CAR). Schematic cross‐sectional view showing the extent of DP‐CAR, and estimated postoperative survival for patients with DP‐CAR. This figure was referred from reference # 26 by Hirano et al

The first English article about DP‐CAR was published by Mayumi and Nimura et al and they operated on six cases with en bloc resection of the celiac artery for carcinoma of the body and tail of the pancreas. Among the six cases, the stomach was preserved in four cases without any severe complications and no mortality.^25^ Moreover, Hirano et al demonstrated long‐term survival data with tremendously better results.^26^ Since they published the excellent surgical outcomes of DP‐CAR (Figure 5), this special operation was highlighted to the pancreatic surgeons in the world. We have followed this advanced operation and figured out the following clinical problems.: first, there is a high incidence of morbidity including delayed gastric emptying, pancreatic fistula, and second, there is poor long‐term survival.^27^, ^28^ To overcome these clinical problems, RCT has been performed to compare pancreaticojejunostomy versus staple closure of the pancreatic stump by multi‐institutions, and anastomosis of jejunum and pancreatic stump had lower incidence of pancreatic fistula if the cut‐end of the pancreas was thicker than 12 mm.^29^ Moreover, we can preserve the left gastric artery in DP‐CAR if the distance from the root of the celiac axis and left gastric artery is more than 2 cm, and low incidence of gastric ischemia and delayed gastric emptying in such patients,^30^ or the left gastric artery should be anastomosed with middle colic artery to maintain the blood flow to the stomach and we expect no risk for gastric ischemia.^31^

### 3.5 How to prevent postoperative complications after pancreaticoduodenectomy

The most serious complication after pancreaticoduodenectomy is pancreatic fistula. To prevent pancreatic fistula, much attention has focused on developing new surgical procedures. Among them, a simple question has been raised, whether early removal of the prophylactic drain will have less incidence of postoperative complications including pancreatic fistula and intraabdominal abscess. To clarify this clinical question, we have performed a prospective study of which is the better procedure, comparing early vs late removal prophylactic drain. We have clearly demonstrated that early removal of the prophylactic drain had much less incidence of pancreatic fistula and intraabdominal abscess.^32^ Following this Japanese trial, Italian groups have conducted a RCT with similar results to Wakayama's trial.^33^ The next attempt to reduce the incidence of pancreatic fistula should be the insertion of a pancreatic stent into the pancreaticojejunostomy. By Motoi and Unno from Sendai, an excellent RCT has been reported about the comparison of external stent drainage vs no stent in patients with soft pancreas, a risk factor of postoperative pancreatic fistula, and they clearly showed the superiority of external stent drainage (pancreatic fistula rate; 10% vs 40%).^34^ Next, the RCT comparing external vs external stent drainage has been reported from Wakayama and showed no difference in the incidence of pancreatic fistula and shorter hospital stay if the patients received the internal stent. Thus, we conclud that internal stent should be one of the options for stent drainage.^35^

**Figure 6:**
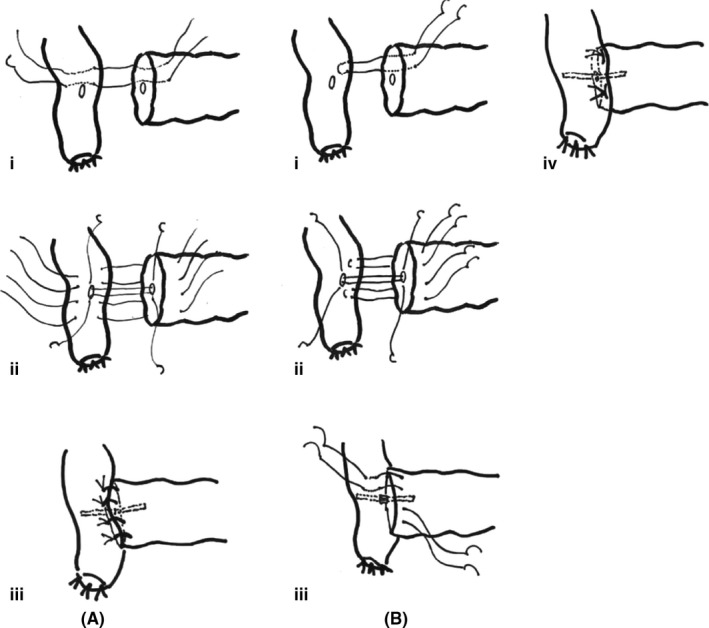
A, Kakita's interrupted suture, and (B) modified Blumgart mattress suture. This figure was referred from reference # 36 by Hirono et al

Recently, several studies reported the superiority of U‐shaped mattress suture proposed by Blumgart compared with the conventional surgical procedures including Kakita method. However, as these studies were retrospective, one could not answer which procedure is superior in terms of the incidence of pancreatic fistula, then we have conducted such RCT comparing conventional Kakita's interrupted suture and modified Blumgart mattress suture in Wakayama Medical University (Figure 6). The results of this RCT revealed that mattress suture of pancreas and jejunum did not reduce clinically relevant pancreatic fistula compared with interrupted suture.^36^ However, the area of fluid collection between pancreas and jejunum was much smaller in mattress suture, that means more tight anastomosis in mattress suture, and, moreover, the costs of sutures during pancreaticojejunostomy were significantly lower in the Blumgart method;^36^ therefore, we prefer to use modified Blumgart mattress sutures in pancreaticojejunostomy. 

## 4 CONCLUSION AND FUTURE PERSPECTIVES

In this review article, the history of pancreatic surgery in Japan is recounted in a retrospective review, allowing us to understand the advances of the Japanese pioneers of pancreatic surgery. Since Fortner et al have reported a special surgery named “regional pancreatectomy,” many Japanese surgeons followed this aggressive surgery, and the survival outcomes were unfortunately dismal due to the early recurrence of metastatic lesions. However, with the recent advances of chemotherapeutic agents, the treatment strategy has changed dramatically.

In the era of newly developed chemotherapeutic agents, one should reconsider the oncological benefits of Fortner's regional pancreatectomy with concomitant perioperative chemotherapy, and other forgotten treatment strategies should be revisited if we are to develop treatment tools. Moreover, the minimally invasive surgeries, including laparoscopic and robotic surgery, are being developed by Japan’s new generation of young pancreatic surgeons. 

Thus, we must inherit the passion and mentality of the Japanese pioneers of pancreatic surgery to develop safer and more secure surgical techniques to reduce morbidities and elongate the survival of pancreatic cancer patients.

## CONFLICT OF INTEREST

The author declares no conflict of interests for this article.
